# The association between pubertal timing and diurnal cortisol activity in middle adolescence

**DOI:** 10.1080/10253890.2026.2718133

**Published:** 2026-08-15

**Authors:** Ellie Roberts, Eirini Flouri

**Affiliations:** a Division of Biosciences, Medical Sciences Building, University College London, London, UK; b Department of Psychology and Human Development, Institute of Education, University College London, London, UK

**Keywords:** Puberty, cortisol, stress, adolescence, gender, ALSPAC

## Abstract

The pubertal development stage is associated with mental health. One explanation is that the pubertal stage is linked to changes in hypothalamic‒pituitary‒adrenal (HPA) axis activity. However, no study has investigated the prospective association between pubertal timing and HPA axis activity, measured by cortisol, in middle adolescence. Using data from 914 individuals (54.70% female) from the Avon Longitudinal Study of Parents and Children (ALSPAC), the current study sought to determine whether pubertal timing across the ages of 8–15.5 years (measured by menarche for girls and voice breaking for boys) was associated with diurnal cortisol slope, the cortisol awakening response, total morning cortisol, and total daily cortisol output at the age of 15.5 years. In girls, earlier menarche was associated with higher (logged) cortisol awakening response (b = −.003; 95% CI = −.007, −.0004), but the association was significant only after adjustment for confounders, especially ethnicity. No associations were observed in boys.

## LAY SUMMARY

Puberty is a time of very significant biological and social changes. This study looked at whether the timing of puberty affects cortisol activity – a hormone involved in the body’s stress response – in middle adolescence. Data from 914 participants were used to investigate whether the timing of puberty affects cortisol activity later, in middle adolescence. Pubertal timing was measured between ages 8 and 15.5 years, using the age of first period in girls and the age of voice breaking in boys. At the age of 15.5, participants provided saliva samples to measure four cortisol outcomes: daily slope (change throughout the day), awakening response (a morning peak), total morning output, and total daily output. The findings showed that girls who started their periods earlier had a higher cortisol awakening response, meaning a sharper rise in cortisol shortly after waking, in middle adolescence. No associations were observed in boys. These results suggest that early-maturing girls exhibit an increased cortisol awakening response in middle adolescence.

## 1. Introduction

Pubertal stages reflect the progression of physical changes throughout adolescence, such as breast development, pubic hair growth, and menstruation for girls and genital development, pubic hair growth, and voice-breaking for boys. The onset of menstruation and voice-breaking typically occurs at the later stages of puberty (Dorn & Biro, 2011). The pubertal development stage has been found to predict a range of concurrent psychological outcomes, with later stages being associated with tiredness, delinquency, irritability, substance use, and somatic complaints (Oldehinkel et al., 2011). More advanced pubertal stages have also been associated with increases in self-reported anxiety levels (Baker et al., 2026) and in fear-based learning, which is prospectively linked to heightened anxiety (Stenson et al., 2021). These associations are found to be more related to physical maturation than age (Baker et al., 2026; Oldehinkel et al., 2011), demonstrating the unique role of puberty.

At the same time, some associations between pubertal stage and psychological outcomes also vary significantly by sex, with girls displaying increases in social uncertainty, depression, and worries (Lewis et al., 2018), and boys showing a decrease in self-criticism. The greater risk of internalizing problems for early-maturing girls has been variously documented, with a recent study showing that, unlike pubertal timing based on visible physical characteristics on the Tanner Stage Line Drawings, timing based on hormone levels was not associated with internalizing psychopathology in girls (Barendse et al., 2022). This, in turn, suggests the importance of psychosocial mechanisms over biological ones in explaining this sex difference. Recently, a systematic review concluding that more advanced pubertal stages are associated with depression in girls but not boys (Stumper & Alloy, 2023) also pointed to the importance of considering sex-specific psychological pathways in explaining this link. For example, alexithymia has been found to mediate the association between pubertal stage and depressive and anxiety symptoms, but only in girls (van der Cruijsen et al., 2019).

The nonpsychosocial mechanisms by which puberty may produce changes in mental health have also received much attention. One pathway may be the coupling of the hypothalamic‒pituitary‒adrenal (HPA) axis, the activation of which causes the secretion of glucocorticoids such as cortisol, and the hypothalamic‒pituitary‒gonadal (HPG) axis, with evidence of crosstalk and additive contribution to mental health problems in adolescence (Marceau et al., 2015). The onset of puberty is associated with a decline in GABA activity, triggering the activation of gonadotropin-releasing hormone (GnRH) (Ojeda et al., 2010), which in turn induces changes in HPG axis function and the secretion of gonadal steroids such as oestradiol and testosterone (Grumbach, 2002). That is, puberty is associated with elevated levels of both sex hormones and cortisol, a stress hormone, which is in turn linked to mental and physical health functions. For example, a review by Adam et al. (2017) reported that flatter diurnal cortisol slopes (i.e. milder changes in cortisol levels throughout the day) are linked to poorer health, including metabolic and cardiovascular diseases, obesity, cancer, fatigue, depression, and anxiety. Particularly in youth, dysregulated cortisol activity can impair emotional stability and cognitive function, with associated structural changes, such as atrophy, in stress-sensitive brain regions, including the hippocampus and amygdala (George et al., 2025).

With cortisol released in response to stressors, it is also important to note that stressor exposure is generally elevated in adolescence (Moskow et al., 2016). During puberty, therefore, increases in stress levels may be both due to the greater exposure to psychosocial stressors, such as more complex peer relationships (Pfeifer & Allen, 2021) and due to alterations in hormone responses, as exposure to gonadal hormones modulates the levels of stress hormones in brain regions implicated in HPA axis reactivity (Romeo, 2010; Ruttle et al., 2015), as explained. Crucially, these mechanisms also interact, whereby experiences and hormonal changes can jointly influence HPA axis reactivity (Romeo, 2010). The pubertal transition has indeed been found to jointly increase cortisol activity (including heightened cortisol awakening response [CAR]) and stressful and social experiences (Reid et al., 2021).

Associations between the pubertal stage and cortisol are now well-established. For example, late pubertal stage individuals have higher total morning cortisol levels than prepubertal individuals (Tsai et al., 2013), as well as higher baseline cortisol and cortisol reactivity (Gunnar et al., 2009; Muscatello & Corbett, 2018). While previous studies, however, have generally demonstrated cross-sectional associations between pubertal development stage and cortisol, it is less clear how specific aspects of puberty, such as timing, may be relate to cortisol activity patterns later in adolescence. One study has found that attenuated cortisol was associated with accelerated pubertal tempo in girls (Saxbe et al., 2015), but no study has investigated whether pubertal timing may be related to cortisol activity later in adolescence. The current study subsequently used a population-representative cohort to investigate whether pubertal timing predicts salivary cortisol activity (diurnal cortisol slope, cortisol awakening response, total morning cortisol, and total cortisol daily output) in girls and boys in middle adolescence.

## 2. Methods

### 2.1. Participants

Data were drawn from the Avon Longitudinal Study of Parents and Children (ALSPAC), which investigates factors affecting development and well-being (Boyd et al., 2013) (see http://www.bristol.ac.uk/alspac/researchers/our-data/ for details). Originally, 14,541 pregnant women in Bristol, UK, were enrolled in the study. From the first trimester, mothers and fathers completed questionnaires about themselves and their child (Northstone and Ben Shlomo, 2023). Children attended clinics for interviews and tests from age 7 (Fraser et al., 2013). In total, 13,988 children were alive at 12 months, and their development has since been followed (Golding, 2004). When the oldest children were aged 7, an attempt was made to increase the sample size. Therefore, the number of new pregnancies not in the initial sample that are currently represented in the released data and reflecting enrollment status at age 30 is 906, resulting in an additional 1,014 children being enrolled. The total sample size for analyses using any data collected after the age of 7 years is therefore 15,454 pregnancies, resulting in 14,901 children alive at 12 months of age (Fraser et al., 2013). Of the initial pregnancies, 338 were women who were already enrolled in a previous pregnancy. Therefore, 14,203 unique mothers were originally enrolled. A further 630 women who did not enroll originally have provided data since their child was 7 years of age. This provides a total of 14,833 unique women (https://www.bristol.ac.uk/alspac/researchers/our-data/). Ethical approval for the study was obtained from the ALSPAC Ethics and Law Committee and the Local Research Ethics Committees. Informed consent for the use of all collected data was obtained from the participants following the recommendations of the ALSPAC Ethics and Law Committee at the time. Participants can contact the study team at any time to retrospectively withdraw consent for their data to be used. Study participation is voluntary, and during all data collection sweeps, information was provided on the intended use of data (further details can be found at https://www.bristol.ac.uk/alspac/researchers/research-ethics/). The completion of a questionnaire, either on paper or online, was considered to be written consent from participants to use their data for research purposes. Biological samples were collected in accordance with the Human Tissue Act (2004). Specific Research Ethics Committee approval is sought for the consenting process at each collection sweep. Written consent, including permission for future use, is obtained from adult participants or from the parents of children as appropriate. Ethical approval for future use is covered by ALSPAC's Research Tissue Bank approval. All historical consents to hold biological samples have been reviewed as part of the Tissue Bank approval process. Participants can contact the study team at any time to retrospectively withdraw consent for use of their samples.

At the age of about 15 years, 5,501 of the ALSPAC cohort children were recruited for a clinical assessment. Of those, 3,020 were invited at random to participate in the cortisol data collection, and 1,845 agreed. After excluding twins, the sample for this study included first borns with at least one valid saliva sample (*N* = 914; 500 girls and 414 boys), representing 6.2% of the total ALSPAC cohort.

### 2.2. Measures

#### 2.2.1. Pubertal timing

Pubertal timing was measured as the age at the first menstrual cycle for girls and voice breaking for boys, following an established approach for deriving pubertal timing in the ALSPAC (Cheng et al., 2022). The ALSPAC administered five questionnaires assessing pubertal development from age 8 to 17 years (average ages of 8, 9.5, 15.5, 16, and 17 years). The responses were capped at 15.5 years (when cortisol was measured) for the main analysis. The measures were completed by caregivers until the participants were 14 years old. For girls, the question was whether they had started their menstrual periods yet, with responses of 0 (no) or 1 (yes). For boys, it was unclear whether their voice had changed yet, with responses of 1 (no), 2 (a little), and 3 (completely). Voice breaking was defined as the age at which the voice broke partially or completely. For respondents whose reports followed a consistent sequential order (i.e. “yes” after “no”), timing was calculated as the midpoint between the last “no” and the first “yes”. If the preceding wave before a “yes” was missing, 12 months were subtracted from the age at the timing report (average interval between the first “yes” and the previous report). If two preceding waves were missing, 18 months were subtracted from the age at the timing report (average interval between the first “yes” and previous report plus half the midpoint interval between average ages at each report for each additional missing report). A sensitivity analysis included those with timing reports after 15.5 years. If pubertal timing was never reported from the age of 8–17 years, 6 months were added to the age range of the last report to estimate timing. Such methods of interval censoring are common in epidemiology, including research on puberty specifically (Biro et al., 2018; Busch et al., 2019). A total of 128 participants had missing data at any preceding waves; therefore, 86% of the sample had complete data on these pubertal measures.

#### 2.2.2. Cortisol activity

Salivary cortisol measurements were taken daily for three consecutive school days at age 15.5 years: at wake-up, 30 min post-wake-up, mid-afternoon, and before bedtime. The participants were instructed to record the sampling times and to place the samples in the freezer until returning them to the laboratory. Each participant agreeing to take part in the study was given a cortisol sampling pack during their clinic visit. Each pack contained detailed sampling instructions, 12 Salivette collection devices (Sarstedt, Germany), a sample collection diary sheet, and a pre-paid envelope in which to return the samples to the laboratory. The participants were instructed to avoid eating and brushing their teeth for at least 30 min prior to sample collection. A time log was given to the participants to record the date and time of each collection. Phone calls were made to participants to remind them to return the samples. Individual samples were inspected for signs of contamination, and time logs were checked for completeness and correspondence to intended sampling times. The samples were then stored at −20°C until being assayed using an enzyme immunoassay (Salimetrics, UK). Inter-assay and intra-assay variation coefficients were 7.9% and 8.9%, respectively. Values that were undetectable, greater than 82 nmol/L, or larger than 4 standard deviations above the sample mean for that time point were treated as missing.

Four cortisol measures were derived: (1) diurnal cortisol slope (difference between mean morning and bedtime cortisol levels divided by hours); (2) cortisol awakening response (CAR) (wake-up cortisol subtracted from post-awakening cortisol); (3) total morning cortisol (sum of the two morning samples); and (4) total daily cortisol output (area under the curve with respect to ground (AUCg) of four time points based on the trapezoid formula) (Pruessner et al., 2003). If the first sample was taken after 10.00 a.m., or the second sample was < 20 or > 60 min after the first, values were treated as missing for that day. Absent or negative CAR values were also treated as missing (*N* = 79), in case of sampling error or non-adherence. Each measure was calculated as the mean of available samples over the study period. Counts for each salivary cortisol sample have been reported elsewhere (Roberts et al., 2026).

Values were log-transformed to correct skewness. Inspection of Pearson kurtosis values and skewness of the cortisol variables was conducted before and after log-transformation. The approximate 95% reference ranges are expected to be under normality and calculated according to the sample size for each cortisol measure (kurtosis values are centered on 3.00, the expected value for a normal Pearson distribution). For the diurnal slope, the kurtosis changed from 12.14 to 6.86 (reference range = 2.67–3.33). The total morning output kurtosis changed from 15.96 to 4.92 (reference range = 2.68–3.32), and skewness from 2.44 to -0.138 (reference range = −0.161–0.161). The AUCg kurtosis changed from 72.06 to 8.17 (reference range = 2.67–3.33), and skewness from 5.97 to 1.15 (reference range = −0.167–0.167). The CAR kurtosis changed from 6.69 to 3.73 (reference range = 2.64–3.36). Log-transformation substantially reduced kurtosis and skewness across cortisol variables, although some deviations from normality remained, particularly for AUCg and the diurnal slope. Owing to the large sample size potentially increasing sensitivity to modest deviations, inspection of Q–Q plots for each cortisol outcome was conducted following linear regression analysis and showed that all the residuals approximated a normal distribution.

### 2.2.3. Confounders

Confounders associated with both pubertal timing and cortisol activity were controlled for, including ethnicity (minority status has been associated with flatter diurnal slope and earlier maturation (Deer et al., 2018; Duncan Cance & Ennett, 2012), body mass index (BMI) (higher values have been associated with earlier maturation and greater cortisol reactivity (Brix et al., 2020; Hewagalamulage et al., 2016), and maternal education and paternal social class (lower socioeconomic status has been associated with earlier pubertal onset and steeper diurnal slope (Ren et al., 2025; Rocha et al., 2022). Ethnicity was coded as white or non-white. BMI was measured at age 15. Maternal education (at 32 weeks of pregnancy) was recorded as Certificate of Secondary Education (CSE, basic school-leaving qualification), vocational/occupation-related training (trade certification), O level/GCSE (high school completion), A level (high school/college diploma), or university/bachelor’s degree, with higher scores representing higher levels of education. Paternal social class (at 32 weeks of pregnancy) was recorded as I, II, III (non-manual), III (manual), IV, or V, with higher scores representing lower levels of social class. Maternal education and paternal social class are frequently used jointly in the ALSPAC to measure family-level socioeconomic position in that sample.

### 2.3. Analytic strategy

Analysis was conducted in STATA 18. Descriptive statistics summarizing sample characteristics. Linear regression analysis was conducted to determine the association between pubertal timing (from ages 8 to 15.5 years) and cortisol activity (at age 15.5 years) in girls and boys, before and after adjustment for confounders. Sensitivity analysis was carried out using all pubertal timing data as predictors in the regression model (and grouping them, after inspecting the histogram of values, as ≤ 12.5 years, 12.6–15.5 years, > 15.5 years), using the youngest group as the reference group (to account for the potential for reverse causality). In all models, missing data were handled with full-information maximum likelihood (FIML). Models were fitted both in complete cases and after FIML.

## 3. Results

### 3.1. Descriptive statistics

A total of 495 girls and 361 boys had data on puberty. Of those, 474 and 279, respectively, had reached puberty by the age of 15.5 years and were therefore included in the analytic sample. Those excluded from the main analysis because they had reached the pubertal milestones later than age 15.5 years were included along with the analytic sample in a sensitivity analysis (details below). Descriptive statistics by sex are displayed in Table 1. The average age at first menstruation was 155.9 months (13.0 years) and the average age of voice breaking was 164.0 months (13.7 years), considering the whole assessment period. A breakdown of pubertal timing is also shown, with age rounded to 0.5 years. All available ages were used, and, as discussed, 6 months was added to the age of the last report to estimate the timing for those who never reported pubertal timing during the assessment period (8–17 years).

**Table 1.** Descriptive statistics of the sample by sex (*N* = 914).

Variable	*N* (%) or Mean (SD) and range	
	Females (*N* = 500)	Males (*N* = 414)
Age at first menstruation (months)	155.9 (14.1), 114.0–207.0; *N* = 495 (Analytic sample: 154.0 (11.4), 114.0–186.0; *N* = 474)	N/A
Age when voice broke (months)	N/A	164.0 (22.5), 114.5–210.0; *N* = 361 (Analytic sample: 153.9 (13.8), 114.5–186.0; *N* = 279)
*Age at pubertal milestone (years)*		
9.5	<5	8
12.5	391	221
13.0	19	7
13.5	<5	<5
14.5	<5	<5
15.0	26	16
15.5	32	33
16.0	15	24
16.5	<5	12
17.0	5	37
17.5	<5	<5
*Ethnicity*		
White	441 (95.45)	376 (96.91)
Non-white	21 (4.55)	12 (3.09)
Body Mass Index (kg/m^2^)	21.80 (3.59), 14.90–38.97 *N* = 498	21.01 (3.36), 14.70–34.05 *N* = 413
*Maternal education*		
CSE	51 (10.87)	30 (7.63)
Vocational	35 (7.46)	35 (8.91)
O level	168 (35.82)	150 (38.17)
A level	141 (30.06)	114 (29.01)
Degree	74 (15.78)	64 (16.28)
*Paternal social class*		
I	57 (12.98)	54 (14.71)
II	169 (38.50)	149 (40.60)
III (non-manual)	56 (12.76)	38 (10.35)
III manual)	126 (28.70)	91 (24.80)
IV	25 (5.69)	26 (7.08)
V	6 (1.37)	9 (2.45)
Diurnal cortisol slope (DCS)*	.112 (.038), –.053–.201 *N* = 463	.104 (.039), –.120–.204 *N* = 382
Cortisol awakening response (CAR)*	.570 (.343), .004–1.88 *N* = 392	.415 (.287), .001–1.56 *N* = 307
Total morning cortisol (TMC)*	4.61 (0.77), 1.34–7.56 *N* = 483	4.22 (0.73), 0.14–7.37 *N* = 403
Total daily cortisol output (AUCg)*	22.92 (5.51), 5.92–61.43 *N* = 453	20.75 (4.29), 10.59–45.42 *N* = 372

^*^ All cortisol outcomes are log-transformed.

### 3.2. Regression models

The results following linear regression analysis before and after adjustment for confounders in complete cases and after FIML are shown in Table 2. In both girls and boys, there were no significant associations between pubertal timing and any cortisol measure in the unadjusted analyses. Later pubertal timing in girls was associated with a lower CAR after adjustment for confounders (b = –.003, 95% CI = –.007, –.000, *p* < .05). This was driven by adjustment for ethnicity as well as socioeconomic status. Non-white ethnicity was associated with a higher CAR, and accounting for ethnicity alone caused this observation to arise. (The results following sequential adjustment for confounders can be found in Supplementary Table 1). Therefore, this result appears to be driven by patterns in ethnic minority girls, who showed higher CAR. No associations between pubertal timing and cortisol activity were observed in boys in any of the models.

**Table 2.** Unadjusted and adjusted regression coefficients in complete cases and after FIML (age at first mensuration and voice-breaking up to 15.5 years).

Complete cases												
	Females						Males					
	Unadjusted			Adjusted			Unadjusted			Adjusted		
	b	SE	95% CI	b	SE	95% CI	b	SE	95% CI	b	SE	95% CI
DCS	−.000	.000	−.0005, .0001	−.000	.000	−.001, .0002	.000	.000	−.0003, .0004	.000	.000	−.0003, .0005
	β = −.053			β = .015			β = .033			β = −.055		
	N = 260			N = 387			N = 229			N = 440		
CAR	−.003	.002	−.006, .0003	−.004*	.002	−.008, -.001	−.001	.001	−.003, .001	−.000	.002	−.004, .003
	β = −.090			β = −.131			β = -.027			β = −.020		
	N = 372			N = 330			N = 212			N = 188		
TMC	−.002	.003	−.008, .004	.001	.003	−.005, .008	−.003	.003	−.010, .004	−.002	.004	−.009, .006
	β = −.032			β = .018			β = −.055			β = −.028		
	N = 459			N = 403			N = 274			N = 243		
AUCg	−.013	.024	−.060, .033	.007	.026	−.044, .057	−.015	.021	−.055, .026	−.024	.023	−.068, .021
	β = −.028			β = .013			β = −.045			β = −.072		
	N = 432			N = 380			N = 255			N = 225		

FIML												
	Females						Males					
	Unadjusted			Adjusted			Unadjusted			Adjusted		
	b	SE	b	SE	b	SE	b	SE	b	SE	b	SE
DCS	−.000	.000	−.0005,	−.000	.000	−.001, .0001	.000	.000	−.0003, .0004	.000	.000	−.0003, .0004
	β = −.055		.0001	β = −.061			β = .016			β = .021		
CAR	−.003	.002	−.006, .0003	−.003*	.002	−.007, -.0004	−.001	.001	−.004, .002	−.000	.002	−.003, .003
	β = −.093			β = −.117			β = −.029			β = -.013		
TMC	−.002	.003	−.008, .004	−.002	.003	−.009, .004	−.003	.003	−.009, .003	−.002	.003	−.008, .004
	β = −.032			β = −.033			β = −.055			β = -.040		
AUCg	−.013	.023	−.059, .033	−.009	.024	−.056, .037	−.014	.019	−.052, .024	−.018	.020	−.057, .021
	β = −.027			β = -.019			β = −.046			β = -.061		

β: Standardized regression coefficient.

### Sensitivity analysis

A sensitivity analysis was conducted to investigate the association between pubertal timing and cortisol activity at age 15.5 years, including those who reached our pubertal milestones after age 15.5 years. Timing was stratified into three categories: (i) earlier than or equal to 12.5 years, (ii) 12.6 to 15.5 years, and (iii) later than 15.5 years (F, *N* = 29; M, *N* = 83, because of rounding, as explained). The youngest group was used as the reference group. The results are shown in Table 3.

No associations between pubertal timing and cortisol activity were observed in this analysis.

**Table 3.** Unadjusted and adjusted regression coefficients in the sample, including those reaching puberty after age 15.5 years, with pubertal timing stratified, in complete cases and after FIML.

Complete cases												
Unadjusted												
	Females (12.6-15.5 years)			Females (>15.5 years)			Males (12.6-15.5 years)			Males (>15.5 years)		
	N = 183			N = 29			N = 102			N = 83		
	Reference group: Females (≤12.5 years) N = 283						Reference group: Males (≤12.5 years) N = 176					
	b	SE	95% CI	b	SE	95% CI	b	SE	95% CI	b	SE	95% CI
DCS	−.002	.004	−.009, .005	.008	.008	−.007, .024	.007	.005	−.003, .017	.008	.005	−.003, .018
(F N = 458)												
(M N = 334)												
CAR	.007	.037	−.065, .080	−.123	.078	−.277, .031	.026	.041	−.055, .107	−.002	.044	−.090, .086
(F N = 387)												
(M N = 271)												
TMC	−.066	.074	−.212, .080	.055	.155	−.251, .360	−.091	.095	−.278, .095	−.024	.101	−.223, .175
(F N = 478)												
(M N = 354)												
AUCg	−.194	.546	−1.27, .879	−1.49	1.20	−3.85, .873	−.271	.558	−1.37, .827	−.695	.609	−1.89, .503
(F N = 448)												
(M N = 325)												

Adjusted												
	Females (12.6-15.5 years)			Females (>15.5 years)			Males (12.6-15.5 years)			Males (>15.5 years)		
	b	SE	95% CI	b	SE	95% CI	b	SE	95% CI	b	SE	95% CI
DCS	−.002	.004	−.010, .006	.009	.009	−.008, .026	.008	.005	−.003, .019	.009	.006	−.003, .020
(F N = 401)												
(M N = 294)												
CAR	−.021	.039	−.098, .055	−.123	.080	−.280, .035	.027	.045	−.062, .115	.007	.048	−.087, .101
(F N = 343)												
(M N = 240)												
TMC	−.037	.079	−.192, .119	.003	.165	−.321, .328	−.088	.105	−.294, .118	−.050	.111	−.269, .168
(F N = 418)												
(M N = 314)												
AUCg	−.179	.588	−1.33, .977	−1.71	1.28	−4.23, .815	−.658	.600	−1.84, .524	−.813	.648	−2.09, .463
(F N = 393)												
(M N = 288)												

FIML												
Unadjusted												
	Females (12.6-15.5 years)			Females (>15.5 years)			Males (12.6-15.5 years)			Males (>15.5 years)		
	b	SE	95% CI	b	SE	95% CI	b	SE	95% CI	b	SE	95% CI
DCS	−.002	.004	−.009, .005	.008	.008	−.007, .024	.007	.005	−.003, .017	.008	.005	−.003, .018
CAR	.007	.037	−.064, .079	−.123	.078	−.275, .030	.027	.041	−.054, .107	−.002	.045	−.089, .085
TMC	−.066	.074	−.210, .079	.054	.154	−.247, .356	−.087	.090	−.263, .089	−.023	.096	−.211, .165
AUCg	−.194	.543	−1.26, .870	−1.48	1.19	−3.82, .857	−.273	.561	−1.37, .826	−.700	.611	−1.90, .498

Adjusted												
	Females (12.6-15.5 years)			Females (>15.5 years)			Males (12.6-15.5 years)			Males (>15.5 years)		
	b	SE	95% CI	b	SE	95% CI	b	SE	95% CI	b	SE	95% CI
DCS	−.002	.004	−.010, .005	.008	.008	−.007, .024	.007	.005	−.003, .016	.007	.005	−.004, .018
CAR	−.001	.036	−.072, .069	−.115	.077	−.265, .035	.033	.042	−.049, .115	.000	.045	−.087, .087
TMC	−.068	.073	−.212, .076	.049	.154	−.253, .352	−.094	.091	−.272, .084	−.043	.096	−.231, .145
AUCg	−.168	.544	−1.23, .898	−1.43	1.20	−3.78, .923	−.375	.568	−1.49, .738	−.785	.615	−1.99, .420

To check for the robustness of our significant finding in the main analysis with regard to CAR, we carried out further sensitivity analyses. The results from models including only those with complete puberty data are shown in Supplementary Table 2, where the findings did not hold, likely due to low power. Models utilizing all CAR values (including absent or negative responses) are displayed in Supplementary Table 3, demonstrating that the findings held. To account for the potential effect of residual differences in the pubertal stage at cortisol assessment on CAR variation, models adjusting for pubertal stage at cortisol assessment are shown in Supplementary Table 4, revealing that consideration of concurrent pubertal stage did not undermine the finding.

## 4. Discussion

The current study sought to investigate whether pubertal timing, as measured by age (across 8–15.5 years) at which the pubertal milestones of menarche and voice-breaking were reached, predicts later cortisol activity (at age 15.5 years), using a population-representative birth cohort in England. In girls, later pubertal timing was associated with a lower cortisol awakening response (CAR). No associations were observed in boys. The finding that later pubertal timing is associated with a lower CAR in girls (i.e. that earlier timing is associated with a higher CAR) builds on previous research reporting that, compared to their counterparts, girls who had reached menarche before starting secondary school had higher salivary cortisol levels and increased depressive symptoms (Trépanier et al., 2013). However, the current study provides further insight into particular measures of cortisol activity that are affected. The observation that pubertal timing was related to CAR, but not other cortisol activity indicators, suggests that the link may not reflect a generalized HPA axis dysfunction in early-maturing girls, but rather a heightened situational response during periods of transition and increased anticipatory stress. CAR has attracted interest as a biomarker of stress reactivity, and has been hypothesized to reflect anticipatory stress, with correlations, for example, shown with perseverative cognition (i.e. rumination and worry) (Zoccola et al., 2011). In our study, ethnic minority girls showed heightened CAR, and only after controlling for ethnicity was a link observed between early menarche and a higher CAR in middle adolescence. The increased likelihood of ethnic minority groups experiencing discrimination and environmental stress exposure (Obomsawin et al., 2025) may contribute to this suppression effect.

Why earlier pubertal timing was associated with HPA axis activity in girls was not explored here, but a much-discussed mechanism may be that, particularly among girls, early timing increases stressors as well as one’s vulnerability to them. Early maturation is related to increased internalizing symptoms (Natsuaki et al., 2009), and puberty coincides with an increase in stressors, such as friendship and school changes, greater academic pressure, and the development of romantic relationships (Caspi & Moffitt, 1991). The combined effects of early menarche and these additional challenges may increase stress (Caspi & Moffitt, 1991), which could explain the elevation in CAR observed here. A heightened CAR has been demonstrated to correlate with internalizing symptoms (Chong et al., 2017) and is predictive of depression onset (Dedovic & Ngiam, 2015), possibly as a marker of anticipatory stress, as discussed. Together, these findings and ours could suggest that puberty is related to such stress, in turn pointing to the importance of targeting anticipatory stress in early-maturing girls.

However, there was no evidence for an association between the timing of puberty and cortisol activity in boys. Puberty is typically found to affect girls more negatively than boys, with girls reporting an increase in mental health difficulties, while boys may even experience a reduction in later pubertal stages (Oldehinkel et al., 2011). These associations have also been demonstrated physiologically, with girls’ baseline cortisol levels increasing over puberty and boys’ declining (Stroud et al., 2011). The sex-specificity of these associations could be related to girls’ greater sensitivity to social evaluation, which may be exacerbated by pubertal changes. This proposition is consistent with previous research demonstrating that increased internalizing problems in early-maturing girls are partly attributed to heightened sensitivity to interpersonal stress (Natsuaki et al., 2009). Another reason for this sex-specificity may be girls’ increased risk of sexual harassment, which is associated with cortisol activity (Trickett et al., 2010), following early maturation. Early-maturing girls may find it more difficult to cope with experiences of sexual harassment and may feel more isolated when handling interactions that do not align with their level of cognitive and emotional development.

Nevertheless, it must be noted that cortisol activity does not represent a direct readout of stress, and no measures of social stress or stressor exposure were included in the current study. Considering that puberty coincides with changes in endocrine regulation, a suggestion that psychosocial stressors represent the predominant underlying mechanism for the effect observed must be considered with caution, as this effect likely encompasses complex interactions between biological and social processes. Still, a review by Kretzer et al. (2024) found that observable physical changes were more predictive than hormonal measures of adolescent mental health, concluding that social processes could be particularly relevant in explaining the link between puberty and mental health. Social processes, particularly social stressors, such as peer victimization, are well-established moderators of the relationship between the pubertal stage and depression (Stumper & Alloy, 2023). Future research should seek to investigate whether such stressors also play a role in the association between pubertal timing and cortisol activity. Another important study limitation is that the sex-specific association with the CAR observed may be due to the weak measurement of pubertal timing for boys. There are important differences in the measurement of pubertal timing for girls and boys in the current study. Voice-breaking is a gradual process compared to menarche, a discrete event. Therefore, the retrospective, mainly parent-reported estimates used are likely more inaccurate for boys.

It is important to consider other study limitations as well. Cortisol has been found to predict pubertal development (Saxbe et al., 2015), but as the current study could not adjust for prior cortisol activity, the possibility of reverse causality in the link between CAR and pubertal timing must be considered. It should also be acknowledged that the effect we found was small. Importantly, since CAR increases over adolescence normatively (Platje et al., 2013), early-maturing individuals may simply have a more “adult-like” CAR before later-maturing individuals. Furthermore, pubertal stage was parent-reported until age 14 years, and therefore, for most participants, pubertal timing was parent-reported. Related to this, the timing of puberty is not exact, as ages were estimated based on average intervals and midpoints in reports, as is common in puberty research (Cheng et al., 2022), but not formally validated. In addition, the sample was predominantly white, which limits its generalizability. This is particularly important as our finding was only significant after adjustment for ethnicity, and likely accentuates the limitation that other factors which influence HPA axis functioning, such as medical conditions and medication/substance use, were not adjusted for. Considering the confounders we did adjust for, our measure of socioeconomic status (maternal education and paternal social class) may seem unusual. It is, however, quite standard in psychiatric epidemiology studies using ALSPAC to capture childhood socioeconomic position in this way (Joinson et al., 2017; Lewis et al., 1998). Maternal education reflects "knowledge capital" and is considered the best proxy for the intellectual environment of the home. Paternal social class reflects "material capital", and in the early 1990s, when the ALSPAC cohort was established, it was the strongest indicator of household income, structural stability, and neighborhood quality. Our method for deriving the diurnal slope was also not standard; therefore, it is uncertain how well this represents a marker of HPA axis function. We were also unable to examine the presence of any baseline differences between the current analytic sample and individuals who were invited to participate in cortisol sampling but did not agree. Finally, these findings may well be historical. ALSPAC is now an aging cohort for puberty studies, given secular trends in pubertal timing. Nevertheless, the study’s longitudinal design enabled the observation of a prospective association between early timing of puberty and increased CAR later in adolescence in girls, which had not been previously demonstrated. Further research should test whether these are causal links, and, if so, explore the mechanisms that may explain them.

## Supplementary Material

Supplementary Material Puberty supplement.docx

## Data Availability

The informed consent obtained from ALSPAC (Avon Longitudinal Study of Parents and Children) participants does not allow the data to be made available through any third-party maintained public repository. Supporting data are available from the ALSPAC on request under the approved proposal number B3681. The full instructions for applying for data access can be found here: http://www.bristol.ac.uk/alspac/researchers/access/. The ALSPAC study website contains details of all available data (http://www.bristol.ac.uk/alspac/researchers/our-data/).

## References

[cit0001] Adam, Emma K. , Quinn, Meghan E. , Tavernier, Royette , McQuillan, Mollie T. , Dahlke, Katie A. , & Gilbert, Kirsten E. (2017). Diurnal cortisol slopes and mental and physical health outcomes: A systematic review and meta-analysis. Psychoneuroendocrinology, *83* , 25–41. 10.1016/j.psyneuen.2017.05.018 28578301 PMC5568897

[cit0002] Baker, A. E. , Akbar, S. A. , Pettit, J. W. , Zhu, A. , Cummings, L. R. , Eihentale, L. , Freitag, J. , Suss, S. J. , Sollenberger, N. A. , Yegüez, C. E. , Rey, Y. , Mattfeld, A. T. , & McMakin, D. L. (2026). A pubertal shift in anxiety reporting: Parent-child discrepancies intensify during peri-adolescence. *Research on Child and Adolescent Psychopathology* , *54* (1), 8. 10.1007/s10802-025-01395-x 41540289 PMC12808191

[cit0003] Barendse, M. E. A. , Byrne, M. L. , Flournoy, J. C. , McNeilly, E. A. , Guazzelli Williamson, V. , Barrett, A. Y. , Chavez, S. J. , Shirtcliff, E. A. , Allen, N. B. , & Pfeifer, J. H. (2022). Multimethod assessment of pubertal timing and associations with internalizing psychopathology in early adolescent girls. *Journal of *Psychopathology* and *Clinical Science* * , *131* (1), 14–25. 10.1037/abn0000721 34941314 PMC9439585

[cit0004] Biro, F. M. , Pajak, A. , Wolff, M. S. , Pinney, S. M. , Windham, G. C. , Galvez, M. P. , Greenspan, L. C. , Kushi, L. H. , & Teitelbaum, S. L. (2018). Age of menarche in a longitudinal US cohort. *Journal of Pediatric and Adolescent Gynecology* , *31* (4), 339–345. 10.1016/j.jpag.2018.05.002 29758276 PMC6121217

[cit0005] Boyd, A. , Golding, J. , Macleod, J. , Lawlor, D. A. , Fraser, A. , Henderson, J. , Molloy, L. , Ness, A. , Ring, S. , & Davey Smith, G. (2013). Cohort profile: The 'children of the 90s'--the index offspring of the avon longitudinal study of parents and children. *International Journal of Epidemiology* , *42* (1), 111–127. 10.1093/ije/dys064 22507743 PMC3600618

[cit0006] Brix, N. , Ernst, A. , Lauridsen, L. L. B. , Parner, E. T. , Arah, O. A. , Olsen, J. , Henriksen, T. B. , & Ramlau-Hansena, C. H. (2020). Childhood overweight and obesity and timing of puberty in boys and girls: Cohort and sibling-matched analyses. *International Journal of Epidemiology* , *49* (3), 834–844. 10.1093/ije/dyaa056 32372073 PMC7394964

[cit0007] Busch, A. S. , Hollis, B. , Day, F. R. , Sørensen, K. , Aksglaede, L. , Perry, J. R. B. , Ong, K. K. , Juul, A. , & Hagen, C. P. (2019). Voice break in boys—temporal relations with other pubertal milestones and likely causal effects of BMI. *Human Reproduction (Oxford, England)* , *34* (8), 1514–1522. 10.1093/humrep/dez118 31348498 PMC6688887

[cit0008] Caspi, A. , & Moffitt, T. E. (1991). Individual differences are accentuated during periods of social change: The sample case of girls at puberty. *Journal of Personality and Social Psychology* , *61* (1), 157–168. 10.1037//0022-3514.61.1.157 1890586

[cit0009] Cheng, T. S. , Sharp, S. J. , Brage, S. , Emmett, P. M. , Forouhi, N. G. , & Ong, K. K. (2022). Longitudinal associations between prepubertal childhood total energy and macronutrient intakes and subsequent puberty timing in UK boys and girls. *European Journal Nutrition* , *61* (1), 157–167. 10.1007/s00394-021-02629-6 PMC8783855 34232374

[cit0010] Chong, L. S. , Thai, M. , Cullen, K. R. , Lim, K. O. , & Klimes-Dougan, B. (2017). Cortisol awakening response, internalizing symptoms, and life satisfaction in emerging adults. *International Journal of Molecular Sciences* , *18* (12), 2501. 10.3390/ijms18122501 29186884 PMC5751104

[cit0011] Dedovic, K. , & Ngiam, J. (2015). The cortisol awakening response and major depression: Examining the evidence. *Neuropsychiatric Disease and Treatment* , *11* , 1181–1189. 10.2147/NDT.S62289 25999722 PMC4437603

[cit0012] Deer, L. K. , Shields, G. S. , Ivory, S. L. , Hostinar, C. E. , & Telzer, E. H. (2018). Racial/ethnic disparities in cortisol diurnal patterns and affect in adolescence. *Development and Psychopathology* , *30* (5), 1977–1993. 10.1017/S0954579418001098 30309395 PMC6345388

[cit0013] Dorn, L. D. , & Biro, F. M. (2011). Puberty and its measurement: A decade in review. *Research on Adolescence* , *21* (1), 180–195. 10.1111/j.1532-7795.2010.00722.x

[cit0014] Duncan Cance, J. , & Ennett, S. T. (2012). Demographic differences in self-report pubertal status among rural adolescents in the US. *Annals of Human Biology* , *39* (1), 84–87. 10.3109/03014460.2011.632647 22092158 PMC4118470

[cit0015] Fraser, A. , Macdonald-Wallis, C. , Tilling, K. , Boyd, A. , Golding, J. , Davey Smith, G. , Henderson, J. , Macleod, J. , Molloy, L. , Ness, A. , Ring, S. , Nelson, S. M. , & Lawlor, D. A. (2013). Cohort profile: The avon longitudinal study of parents and children: ALSPAC mothers cohort. *International Journal of Epidemiology* , *42* (1), 97–110. 10.1093/ije/dys066 22507742 PMC3600619

[cit0016] George, M. Y. , Abdel Mageed, S. S. , Mansour, D. E. , & Fawzi, S. F. (2025). The cortisol axis and psychiatric disorders: An updated review. *Pharmacological Reports : PR* , *77* (6), 1573–1599. 10.1007/s43440-025-00782-x 40956392 PMC12647353

[cit0017] Golding, J. , ALSPAC Study Team . (2004). The avon longitudinal study of parents and children (ALSPAC)--study design and collaborative opportunities. *European Journal of Endocrinology* , *151* (Suppl 3), U119–U123. 10.1530/eje.0.151u119 15554896

[cit0018] Grumbach, M. M. (2002). The neuroendocrinology of human puberty revisited. *Hormone Research* , *57* (Suppl 2), 2–14. 10.1159/000058094 12065920

[cit0019] Gunnar, M. R. , Wewerka, S. , Frenn, K. , Long, J. D. , & Griggs, C. (2009). Developmental changes in hypothalamus-pituitary-adrenal activity over the transition to adolescence: Normative changes and associations with puberty. *Development and Psychopathology* , *21* (1), 69–85. 10.1017/S0954579409000054 19144223 PMC3933029

[cit0020] Hewagalamulage, S. D. , Lee, T. K. , Clarke, I. J. , & Henry, B. A. (2016). Stress, cortisol, and obesity: A role for cortisol responsiveness in identifying individuals prone to obesity. *Domestic Animal Endocrinology* , *56 Suppl* , S112–S120. 10.1016/j.domaniend.2016.03.004 27345309

[cit0021] Human Tissue Act . (2004). UK Public General Acts. https://www.legislation.gov.uk/ukpga/2004/30/contents

[cit0022] Joinson, C. , Kounali, D. , & Lewis, G. (2017). Family socioeconomic position in early life and onset of depressive symptoms and depression: A prospective cohort study. *Social Psychiatry and Psychiatric Epidemiology* , *52* (1), 95–103. 10.1007/s00127-016-1308-2 27837235 PMC5226994

[cit0023] Kretzer, S. , Lawrence, A. J. , Pollard, R. , Ma, X. , Chen, P. J. , Amasi-Hartoonian, N. , Pariante, C. , Vallée, C. , Meaney, M. , & Dazzan, P. (2024). The dynamic interplay between puberty and structural brain development as a predictor of mental health difficulties in adolescence: A systematic review. *Biological Psychiatry* , *96* (7), 585–603. 10.1016/j.biopsych.2024.06.012 38925264 PMC11794195

[cit0024] Lewis, G. , Bebbington, P. , Brugha, T. , Farrell, M. , Gill, B. , Jenkins, R. , & Meltzer, H. (1998). Socioeconomic status, standard of living, and neurotic disorder. *Lancet (London)* , *352* (9128), 605–609. 10.1016/S0140-6736(98)04494-8 9746021

[cit0025] Lewis, G. , Ioannidis, K. , van Harmelen, A. L. , Neufeld, S. , Stochl, J. , Lewis, G. , Jones, P. B. , & Goodyer, I. (2018). The association between pubertal status and depressive symptoms and diagnoses in adolescent females: A population-based cohort study. *PLoS One* , *13* (6), e0198804. 10.1371/journal.pone.0198804 29912985 PMC6005470

[cit0026] Marceau, K. , Ruttle, P. L. , Shirtcliff, E. A. , Essex, M. J. , & Susman, E. J. (2015). Developmental and contextual considerations for adrenal and gonadal hormone functioning during adolescence: Implications for adolescent mental health. *Developmental Psychobiology (New York, NY)* , *57* (6), 742–768. 10.1002/dev.21214 PMC4194172 24729154

[cit0027] Moskow, D. M. , Addington, J. , Bearden, C. E. , Cadenhead, K. S. , Cornblatt, B. A. , Heinssen, R. , Mathalon, D. H. , McGlashan, T. H. , Perkins, D. O. , Seidman, L. J. , Tsuang, M. T. , Cannon, T. D. , Woods, S. W. , & Walker, E. F. (2016). The relations of age and pubertal development with cortisol and daily stress in youth at clinical risk for psychosis. *Schizophrenia Research* , *172* (1-3), 29–34. 10.1016/j.schres.2016.02.002 26905038 PMC4821739

[cit0028] Muscatello, R. A. , & Corbett, B. A. (2018). Comparing the effects of age, pubertal development, and symptom profile on cortisol rhythm in children and adolescents with autism spectrum disorder. *Autism Research* , *11* (1), 110–120. 10.1002/aur.1879 29030905 PMC6453123

[cit0029] Natsuaki, M. N. , Klimes-Dougan, B. , Ge, X. , Shirtcliff, E. A. , Hastings, P. D. , & Zahn-Waxler, C. (2009). Early pubertal maturation and internalizing problems in adolescence: Sex differences in the role of cortisol reactivity to interpersonal stress. *Journal of Clinical Child and Adolescent Psychology : the Official Journal for the Society of Clinical Child and Adolescent Psychology, American Psychological Association, Division 53* , *38* (4), 513–524. 10.1080/15374410902976320 20183638 PMC3061854

[cit0030] Northstone, K. , Ben Shlomo, Y. , Teyhan, A. , Hill, A. , Groom, A. , Mumme, M. , Timpson, N. , & Golding, J. (2023). The avon longitudinal study of parents and children ALSPAC G0 partners: A cohort profile. *Wellcome Open Research* , *37* (8). 10.12688/wellcomeopenres.18782.1

[cit0031] Obomsawin, A. , Pejic, S. R. , & Koziol, C. (2025). Effects of racial discrimination tasks on salivary cortisol reactivity among racially minoritized groups: A meta-analysis. *Hormones and Behavior* , *173* . 105775. 10.1016/j.yhbeh.2025.105775 40482563

[cit0032] Ojeda, S. R. , Lomniczi, A. , Loche, A. , Matagne, V. , Kaidar, G. , Sandau, U. S. , & Dissen, G. A. (2010). The transcriptional control of female puberty. *Brain Research (Amsterdam)* , *1364* , 164–174. 10.1016/j.brainres.2010.09.039 PMC2992593 20851111

[cit0033] Oldehinkel, A. J. , Verhulst, F. C. , & Ormel, J. (2011). Mental health problems during puberty: Tanner stage-related differences in specific symptoms. The TRAILS study. *Journal of Adolescence (London)* , *34* (1), 73–85. 10.1016/j.adolescence.2010.01.010 20172603

[cit0034] Pfeifer, J. H. , & Allen, N. B. (2021). Puberty initiates cascading relationships between neurodevelopmental, social, and internalizing processes across adolescence. *Biological Psychiatry* , *89* (2), 99–108. 10.1016/j.biopsych.2020.09.002 33334434 PMC8494463

[cit0035] Platje, E. , Vermeiren, R. R. , Branje, S. J. , Doreleijers, T. A. , Meeus, W. H. , Koot, H. M. , Frijns, T. , van Lier, P. A. , & Jansen, L. M. (2013). Long-term stability of the cortisol awakening response over adolescence. *Psychoneuroendocrinology* , *38* (2), 271–280. 10.1016/j.psyneuen.2012.06.007 22776421

[cit0036] Pruessner, J. C. , Kirschbaum, C. , Meinlschmid, G. , & Hellhammer, D. H. (2003). Two formulas for computation of the area under the curve represent measures of total hormone concentration versus time-dependent change. *Psychoneuroendocrinology* , *28* (7), 916–931. 10.1016/S0306-4530(02)00108-7 12892658

[cit0037] Reid, B. M. , DePasquale, C. E. , Donzella, B. , Leneman, K. B. , Taylor, H. , & Gunnar, M. R. (2021). Pubertal transition with current life stress and support alters longitudinal diurnal cortisol patterns in adolescents exposed to early life adversity. *Developmental Psychobiology (New York, NY)* , *63* (6. e22146. 10.1002/dev.22146 PMC8815064 34053063

[cit0038] Ren, Y. , Sun, L. , Qiu, S. , Ming, H. , Zhang, Y. , Zuo, C. , Zhou, Y. , Mei, K. , & Huang, S. (2025). The impact of neighborhood and family socioeconomic status on Adolescents' internalizing symptoms: The mediating role of pubertal development trajectory. *Journal Youth Adolescence* , *54* (10), 2695–2711. 10.1007/s10964-025-02247-z 40887531

[cit0039] Roberts, E. , Francesconi, M. , & Flouri, E. (2026). The relationship between childhood autistic traits and diurnal cortisol activity in adolescence. *Psychoneuroendocrinology* , *183* . 107672. 10.1016/j.psyneuen.2025.107672 41176904

[cit0040] Rocha, S. , Almeida, D. M. , Chiang, J. J. , Cole, S. W. , Irwin, M. R. , Seeman, T. , & Fuligni, A. J. (2022). The relationship between family socioeconomic status and adolescent sleep and diurnal cortisol. *Psychosomatic Medicine (Baltimore, MD)* , *84* (7), 848–855. 10.1097/PSY.0000000000001104 PMC9437129 35797448

[cit0041] Romeo, R. D. (2010). Pubertal maturation and programming of hypothalamic–pituitary–adrenal reactivity. *Frontiers in Neuroendocrinology. FNEDA7* , *31* (2), 232–240. 10.1016/j.yfrne.2010.02.004 20193707

[cit0042] Ruttle, P. L. , Shirtcliff, E. A. , Armstrong, J. M. , Klein, M. H. , & Essex, M. J. (2015). Neuroendocrine coupling across adolescence and the longitudinal influence of early life stress. *Developmental Psychobiology (New York, NY)* , *57* (6), 688–704. 10.1002/dev.21138 PMC6585402 23775330

[cit0043] Saxbe, D. E. , Negriff, S. , Susman, E. J. , & Trickett, P. K. (2015). Attenuated hypothalamic-pituitary-adrenal axis functioning predicts accelerated pubertal development in girls 1 year later. *Development and Psychopathology* , *27* (3), 819–828. 10.1017/S0954579414000790 25154521 PMC4342325

[cit0044] Stenson, A. F. , Nugent, N. R. , van Rooij, S. J. H. , Minton, S. T. , Compton, A. B. , Hinrichs, R. , & Jovanovic, T. (2021). Puberty drives fear learning during adolescence. *Developmental Science* , *24* (1. e13000. 10.1111/desc.13000 32497415 PMC9206403

[cit0045] Stroud, L. R. , Papandonatos, G. D. , Williamson, D. E. , & Dahl, R. E. (2011). Sex differences in cortisol response to corticotropin releasing hormone challenge over puberty: Pittsburgh pediatric neurobehavioral studies. *Psychoneuroendocrinology* , *36* (8), 1226–1238. 10.1016/j.psyneuen.2011.02.017 21489699 PMC3270708

[cit0046] Stumper, A. , & Alloy, L. B. (2023). Associations between pubertal stage and depression: A systematic review of the literature. *Child Psychiatry and Human Development* , *54* (2), 312–339. 10.1007/s10578-021-01244-0 34529199 PMC11869324

[cit0047] Trépanier, L. , Juster, R. P. , Marin, M. F. , Plusquellec, P. , Francois, N. , Sindi, S. , Wan, N. , Findlay, H. , Schramek, T. , Andrews, J. , Corbo, V. , Dedovic, K. , & Lupien, S. (2013). Early menarche predicts increased depressive symptoms and cortisol levels in quebec girls ages 11 to 13. *Development and Psychopathology* , *25* (4 Pt 1), 1017–1027. 10.1017/S0954579413000345 24229546

[cit0048] Trickett, P. K. , Noll, J. G. , Susman, E. J. , Shenk, C. E. , & Putnam, F. W. (2010). Attenuation of cortisol across development for victims of sexual abuse. *Development and Psychopathology* , *22* (1), 165–175. 10.1017/S0954579409990332 20102654 PMC3100742

[cit0049] Tsai, S. L. , Seiler, K. J. , & Jacobson, J. (2013). Morning cortisol levels affected by sex and pubertal status in children and young adults. *Journal of Clinical Research in Pediatric Endocrinology* , *5* (2), 85–89. 10.4274/Jcrpe.892 23748059 PMC3701927

[cit0050] van der Cruijsen, Renske , Murphy, Jennifer , & Bird, Geoffrey . (2019). Alexithymic traits can explain the association between puberty and symptoms of depression and anxiety in adolescent females. PLOS ONE, 14, e0210519. 10.1371/journal.pone.0210519 30650139 PMC6334924

[cit0051] Zoccola, P. M. , Dickerson, S. S. , & Yim, I. S. (2011). Trait and state perseverative cognition and the cortisol awakening respons. 10.1016/j.psyneuen.2010.10.004 21050668

