# Supplementary material for: The association between pubertal timing and diurnal cortisol activity in middle adolescence
Source: Stress. 2026 Aug 15;29(1):2718133. doi: 10.1080/10253890.2026.2718133 (PMC13479936; doi:10.1080/10253890.2026.2718133)
Supplement: Supplementary file 1 [file ISTS_A_2718133_SM2047.docx]

**Supplementary material**

**Supplementary Table 1.** Unadjusted and adjusted regression coefficients in complete cases and after FIML with sequential adjustment for confounders (onset up to 15.5 years)

| **Complete cases** | | | | | | |
| --- | --- | --- | --- | --- | --- | --- |
| Unadjusted | | | | | | |
|  | Females | | | Males | | |
|  | b | SE | 95% CI | b | SE | 95% CI |
| DCS  (F N = 440) (M N = 260) | -.000 | .000 | -.0005, .0001 | .000 | .000 | -.0003, .0004 |
| CAR  (F N = 372)  (M N = 212) | -.003 | .002 | -.006, .0003 | -.001 | .001 | -.003, .001 |
| TMC  (F N = 459)  (M N = 274) | -.002 | .003 | -.008, .004 | -.003 | .003 | -.010, .004 |
| AUCg  (F N = 432)  (M N = 255) | -.013 | .024 | -.060, .033 | -.015 | .021 | -.055, .026 |
| Adjusted (ethnicity) | | | | | | |
|  | Females | | | Males | | |
|  | b | SE | 95% CI | b | SE | 95% CI |
| DCS  (F N = 408)  (M N = 248) | -.000 | .000 | -.0005, .0002 | .000 | .000 | -.0003, .0004 |
| CAR  (F N = 347)  (M N = 204) | -.004* | .002 | -.007, -.001 | -.0003 | .002 | -.003, .003 |
| TMC  (F N = 425)  (M N = 262) | .000 | .003 | -.006, .007 | -.003 | .003 | -.010, .004 |
| AUCg  (F N = 401)  (M N = 243) | -.006 | .025 | -.056, .043 | -.010 | .021 | -.052, .032 |
| Adjusted (ethnicity, maternal education, paternal social class) | | | | | | |
|  | Females | | | Males | | |
|  | b | SE | 95% CI | b | SE | 95% CI |
| DCS  (F N = 388)  (M N = 229) | -.000 | .000 | -.001, .0002 | .000 | .000 | -.0003, .0005 |
| CAR  (F N = 331)  (M N = 188) | -.004* | .002 | -.008, -.001 | -.000 | .002 | -.004, .003 |
| TMC  (F N = 404)  (M N = 243) | .001 | .003 | -.005, .008 | -.002 | .004 | -.009, .006 |
| AUCg  (F N = 381)  (M N = 225) | .006 | .026 | -.045, .057 | -.021 | .023 | -.066, .024 |
| Adjusted (ethnicity, maternal education, paternal social class, BMI) | | | | | | |
|  | Females | | | Males | | |
|  | b | SE | 95% CI | b | SE | 95% CI |
| DCS  (F N = 387) (M N = 229) | -.000 | .000 | -.001, .0002 | .000 | .000 | -.0003, .0005 |
| CAR  (F N = 330)  (M N = 188) | -.004* | .002 | -.008, -.001 | -.000 | .002 | -.004, .003 |
| TMC  (F N = 403)  (M N = 243) | .001 | .003 | -.005, .008 | -.002 | .004 | -.009, .006 |
| AUCg  (F N = 380)  (M N = 225) | .007 | .026 | -.044, .057 | -.024 | .023 | -.068, .021 |
| **FIML** | | | | | | |
| Unadjusted | | | | | | |
|  | Females | | | Males | | |
|  | b | SE | 95% CI | b | SE | 95% CI |
| DCS | -.000 | .000 | -.0005, .0001 | .000 | .000 | -.0003, .0004 |
| CAR | -.003 | .002 | -.006, .0003 | -.001 | .001 | -.004, .002 |
| TMC | -.002 | .003 | -.008, .004 | -.003 | .003 | -.009, .003 |
| AUCg | -.013 | .023 | -.059, .033 | -.014 | .019 | -.052, .024 |
| Adjusted (ethnicity) | | | | | | |
|  | Females | | | Males | | |
|  | b | SE | 95% CI | b | SE | 95% CI |
| DCS | -.000 | .000 | -.0005, .0001 | .000 | .000 | -.0003, .0004 |
| CAR | -.003* | .002 | -.006, -.0002 | -.001 | .001 | -.004, .002 |
| TMC | -.002 | .003 | -.008, .004 | -.003 | .003 | -.010, .003 |
| AUCg | -.012 | .024 | -.058, .035 | -.015 | .021 | -.055, .026 |
| Adjusted (ethnicity, maternal education, paternal social class) | | | | | | |
|  | Females | | | Males | | |
|  | b | SE | 95% CI | b | SE | 95% CI |
| DCS | -.000 | .000 | -.0005, .0001 | .000 | .000 | -.0003, .000 |
| CAR | -.003* | .002 | -.007, -.0003 | -.000 | .002 | -.003, .003 |
| TMC | -.002 | .003 | -.008, .004 | -.002 | .003 | -.009, .005 |
| AUCg | -.011 | .024 | -.058, .036 | -.017 | .021 | -.059, .024 |
| Adjusted (ethnicity, maternal education, paternal social class, BMI) | | | | | | |
|  | Females | | | Males | | |
|  | b | SE | 95% CI | b | SE | 95% CI |
| DCS | -.000 | .000 | -.001, .0001 | .000 | .000 | -.0003, .0004 |
| CAR | -.003* | .002 | -.007, -.0004 | -.000 | .002 | -.003, .003 |
| TMC | -.002 | .003 | -.009, .004 | -.002 | .003 | -.008, .004 |
| AUCg | -.009 | .024 | -.056, .037 | -.018 | .020 | -.057, .021 |

**Supplementary Table 2.** Unadjusted and adjusted regression coefficients in complete cases and after FIML (age at first mensuration and voice-breaking up to 15.5 years) for individuals with complete puberty data

| **Complete cases** | | | | | | | | | | | | |
| --- | --- | --- | --- | --- | --- | --- | --- | --- | --- | --- | --- | --- |
|  | Females | | | | | | Males | | | | | |
|  | Unadjusted | | | Adjusted | | | Unadjusted | | | Adjusted | | |
|  | b | SE | 95% CI | b | SE | 95% CI | b | SE | 95% CI | b | SE | 95% CI |
| DCS | -.000  N = 415 | .000 | -.001, .0002 | -.000  N = 362 | .000 | -.001, .0003 | -.001  N = 222 | .000 | -.002, .0002 | -.001  N = 197 | .000 | -.002, .0003 |
| CAR | -.002  N = 352 | .002 | -.006, .002 | -.004  N = 310 | .003 | -.009, .001 | .001  N = 182 | .004 | -.007, .008 | .000  N = 164 | .004 | -.007, .008 |
| TMC | -.000  N = 434 | .004 | -.008, .008 | .007  N = 378 | .005 | -.002, .016 | -.011  N = 232 | .008 | -.026, .004 | -.010  N = 207 | .008 | -.026, .006 |
| AUCg | .018  N = 407 | .030 | -.042, .078 | .062  N = 355 | .036 | -.009, .133 | -.014  N = 217 | .051 | -.114, .086 | -.029  N = 193 | .053 | -.133, .075 |
| **FIML** | | | | | | | | | | | | |
|  | Females | | | | | | Males | | | | | |
|  | Unadjusted | | | Adjusted | | | Unadjusted | | | Adjusted | | |
|  | b | SE | 95% CI | b | SE | 95% CI | b | SE | 95% CI | b | SE | 95% CI |
| DCS | -.000 | .000 | -.001, .0002 | -.000 | .000 | -.001, .0002 | -.001 | .000 | -.002, .0002 | -.001 | .000 | -.002, .0002 |
| CAR | -.002 | .002 | -.006, .002 | -.003 | .002 | -.007, .001 | .001 | .004 | -.007, .008 | .000 | .004 | -.007, .008 |
| TMC | -.000 | .004 | -.008, .008 | -.000 | .004 | -.008, .008 | -.011 | .008 | -.026, .004 | -.011 | .008 | -.026, .004 |
| AUCg | .018 | .030 | -.042, .077 | .021 | .031 | -.040, .081 | -.014 | .050 | -.113, .085 | -.028 | .051 | -.128, .071 |

**Supplementary Table 3.** Unadjusted and adjusted regression coefficients in complete cases and after FIML (age at first mensuration and voice-breaking up to 15.5 years) including all CAR values

| **Complete cases** | | | | | | | | | | | | |
| --- | --- | --- | --- | --- | --- | --- | --- | --- | --- | --- | --- | --- |
|  | Females | | | | | | Males | | | | | |
|  | Unadjusted | | | Adjusted | | | Unadjusted | | | Adjusted | | |
|  | b | SE | 95% CI | b | SE | 95% CI | b | SE | 95% CI | b | SE | 95% CI |
| CAR | -.008  N = 408 | .004 | -.016, .0003 | -.010*  N = 358 | .005 | -.019, -.001 | .002  N = 240 | .004 | -.006, .011 | .004  N = 213 | .005 | -.006, .013 |
| **FIML** | | | | | | | | | | | | |
|  | Females | | | | | | Males | | | | | |
|  | Unadjusted | | | Adjusted | | | Unadjusted | | | Adjusted | | |
|  | b | SE | 95% CI | b | SE | 95% CI | b | SE | 95% CI | b | SE | 95% CI |
| CAR | -.008 | .004 | -.016, .0002 | -.008* | .004 | -.016, -.0004 | .002 | .004 | -.006, .011 | .004 | .004 | -.005, .012 |

**Supplementary Table 4.** Unadjusted and adjusted regression coefficients in complete cases and after FIML (age at first mensuration and voice-breaking up to 15.5 years), all adjusted for pubertal stage at cortisol assessment

| **Complete cases** | | | | | | | | | | | | |
| --- | --- | --- | --- | --- | --- | --- | --- | --- | --- | --- | --- | --- |
|  | Females | | | | | | Males | | | | | |
|  | Unadjusted | | | Adjusted | | | Unadjusted | | | Adjusted | | |
|  | b | SE | 95% CI | b | SE | 95% CI | b | SE | 95% CI | b | SE | 95% CI |
| DCS | -.000  N = 440 | .000 | -.001,  .00009 | -.000  N = 387 | .000 | -.001, .0001 | .000  N = 260 | .000 | -.0001, .0004 | .000  N = 229 | .000 | -.0003, .001 |
| CAR | -.002  N = 372 | .002 | -.006, .001 | -.004*  N = 330 | .002 | -.008, -.0001 | -.001  N = 212 | .001 | -.004, .002 | -.001  N = 188 | .002 | -.004, .003 |
| TMC | -.002  N = 459 | .003 | -.009, .005 | .002  N = 403 | .004 | -.005, .009 | -.004  N = 274 | .003 | -.010, .003 | -.002  N = 243 | .004 | -.010, .006 |
| AUCg | -.003  N = 432 | .025 | -.053, .046 | .021  N = 380 | .028 | -.033, .076 | -.018 N = 255 | .021 | -.059, .023 | -.030  N = 225 | .023 | -.075, .016 |
| **FIML** | | | | | | | | | | | | |
|  | Females | | | | | | Males | | | | | |
|  | Unadjusted | | | Adjusted | | | Unadjusted | | | Adjusted | | |
|  | b | SE | 95% CI | b | SE | 95% CI | b | SE | 95% CI | b | SE | 95% CI |
| DCS | -.000 | .000 | -.001,  .00009 | -.000 | .000 | -.001,  .00007 | .000 | .000 | -.0003, .0004 | .000 | .000 | -.0003, .0004 |
| CAR | -.002 | .002 | -.006, .001 | -.003 | .002 | -.007,  .00006 | -.001 | .001 | -.003, .002 | -.000 | .001 | -.003, .003 |
| TMC | -.002 | .003 | -.009, .004 | -.002 | .003 | -.009, .004 | -.004 | .003 | -.010, .003 | -.003 | .003 | -.009, .004 |
| AUCg | -.003 | .025 | -.052, .046 | .001 | .025 | -.049, .051 | -.018 | .021 | -.058, .022 | -.024 | .021 | -.065, .018 |
